# Fulminant Myocarditis Managed by Extracorporeal Life Support (Impella® CP): A Rare Case

**DOI:** 10.1155/2017/9231959

**Published:** 2017-07-12

**Authors:** Henrik Fox, Martin Farr, Dieter Horstkotte, Christian Flottmann

**Affiliations:** Clinic for Cardiology, Herz- und Diabeteszentrum NRW, Ruhr-Universität Bochum, Bad Oeynhausen, Germany

## Abstract

**Background:**

Treating myocarditis can be difficult, as clear criteria for diagnosis and management are lacking for heterogeneous clinical presentations.

**Case Description:**

We report a case of a 49-year-old female who presented with cardiogenic shock and subsequent cardiac arrest. Extracorporeal life support was instituted, and after eight days with Impella CP the patient recovered and at six months presented with normal cardiac function.

**Conclusion:**

Fulminant myocarditis remains a challenging disease in daily clinical practice, not only for diagnosis, but also for treatment. With this report we emphasize that myocardial failure due to fulminant myocarditis may be reversible if treated with extracorporeal life support, which thus plays an important and life-saving role.

## 1. Introduction

In daily clinical practice, acute myocarditis remains a challenge, as diagnosis may be difficult and is often missed due to its clinical heterogeneity [1]. Clear criteria for diagnosis and management are lacking for the wide and heterogeneous clinical presentations, which complicate patient identification and consensus on the most appropriate treatment [1]. Accepted is the WHO (World Health Organization)/IFC (International Society and Federation of Cardiology/World Heart Federation) definition [1], diagnosed by histological, immunological, and immunohistochemical criteria [2] and Dallas criteria [3].

In cases of fulminant course with cardiogenic shock, extracorporeal life support may be the only treatment to overcome acute heart failure [4]. We report a case of an unusual myocarditis, with the patient surviving through extracorporeal life support.

## 2. Case Report

**Figure 1:**
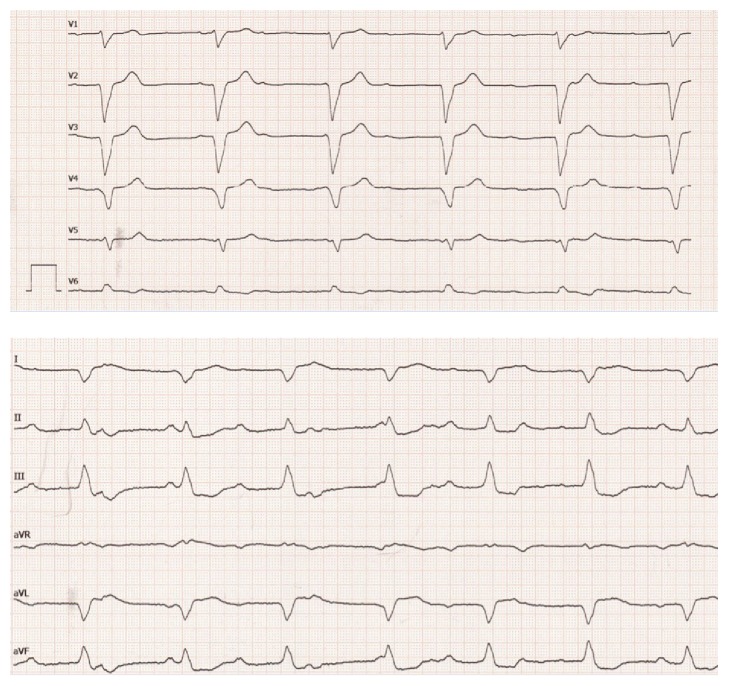
12 lead ECG upon admission to our center.

**Figure 2:**
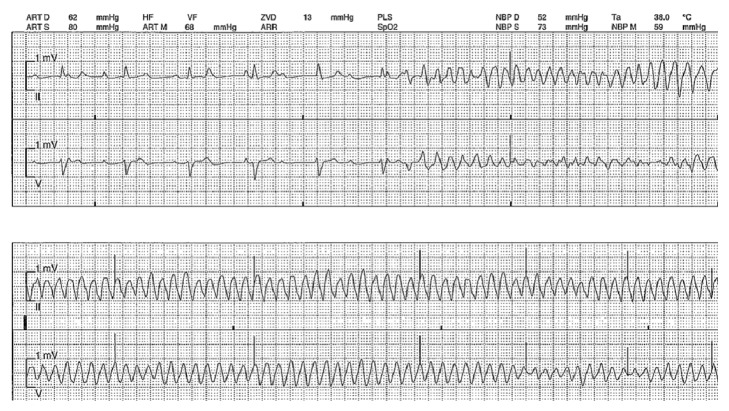
Monitor recording of episode of sudden cardiac arrest.

**Figure 3:**
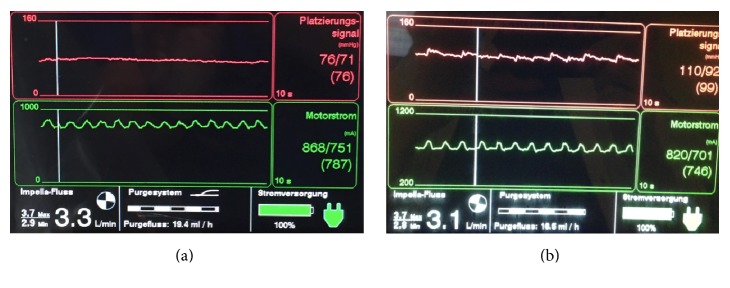
Impella CP Monitor. Impella CP Monitor at initial stage (a); Impella CP Monitor at advanced stage (b).

A 49-year-old female, with no former relevant medical history and no former regular medication, had reported flu-like symptoms with physical weakness, dizziness, headache, and chills. After three days she collapsed and on admission to hospital an advanced cardiogenic shock with initial left ventricular ejection fraction of 10% was seen. Hemodynamic monitoring revealed a cardiac index of 1.8 l/min/m^2^. Arterial blood gas analysis showed serum lactate of 3.7 mmol/l and elevated transaminases (GOT 2624 (0–35) U/l, GPT 2234 (0–35) U/l), cardiac markers (creatine kinase 1730 (0–145) U/l, high sensitive troponin-I 93430 (0–26) pg/ml) were significantly elevated, with no signs of acute myocardial infarction in the ECG (Figure 1). The patient suffered cardiac arrest (Figure 2) and was treated with catecholamines as well as a calcium sensitizer (Levosimendan, Simdax®, Orion Corporation, Espoo, Finland), but for stabilization an Impella CP (Abiomed Inc., Danvers, MA, USA) was necessary (Figure 3).

The patient underwent coronary angiography for exclusion of coronary artery disease and myocardial biopsy for further assessment. Blood and biopsy specimens were tested for common causative agents, such as Coxsackieviruses A and B, echoviruses, polioviruses, influenza A and B viruses, respiratory syncytial virus, mumps virus, measles virus, rubella virus, hepatitis C virus, dengue virus, yellow fever virus, Chikungunya virus, human immunodeficiency virus-1, adenoviruses, parvovirus B19, cytomegalovirus, human herpes virus-6, Epstein-Barr virus, varicella-zoster virus, and herpes simplex virus, as recommended [1], but none of these triggers was detected.

During the following days, left ventricular systolic function improved and after 8 days Impella was explanted. After six months, LVEF was 55%, and the patient lives a normal life.

## 3. Endomyocardial Biopsy

**Figure 4:**
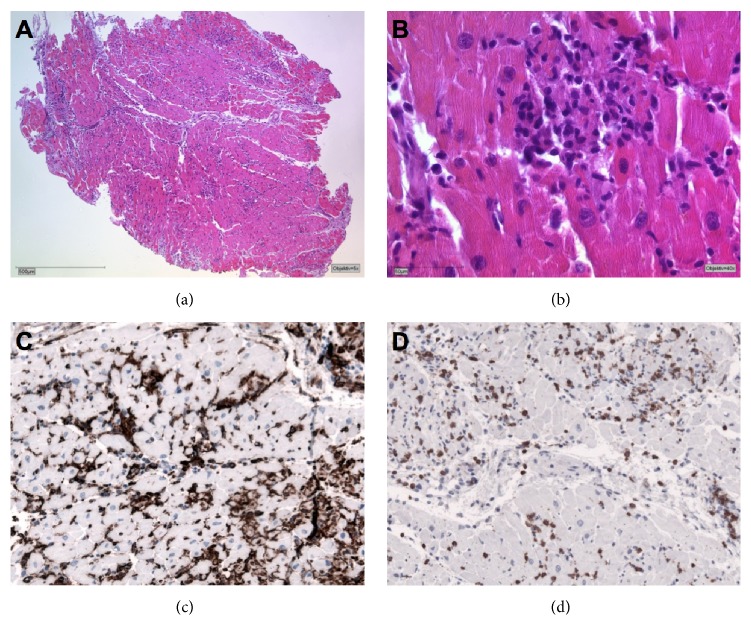
Endomyocardial biopsy. Histological detection of massive infiltration by mononuclear inflammatory cells: (a) overview section (50x); (b) cluster infiltrated immunocompetent cells (purple, 400x), myocardial damage; (c) MHCII-overexpressing immunocompetent cells (esp. macrophages, 200x); and (d) CD3-T-lymphocytes (brown 200x).

Right ventricular endomyocardial biopsy (EMB) was taken. Viral PCR (polymerase chain reaction) in RNA-stabilized EMB and EDTA (ethylenediaminetetraacetic acid) blood demonstrated absence of infectious causative organisms. Histological analysis of formalin-fixed and paraffin-embedded, and hematoxylin/eosin-stained EMB showed infiltration of mononuclear cells, myocardial damage, and interstitial fibrosis (Dallas criteria [1, 3]). Immunohistology detected numerous CD3-positive T-lymphocytes and a number of CD68-positive and MHCII-overexpressing macrophages (Figure 4).

## 4. Discussion

In clinical routine, myocarditis may be missed, as postmortem studies in prospective settings showed frequencies in young adults with sudden cardiac death in 8.6% to 12% of cases [1]. Facilities for diagnosis have advanced in recent years, as molecular techniques allow a more precise insight into inflammatory autoimmune processes [1]. Morbidity and mortality of myocarditis is well known, while the broad variety of etiologies impede universal recommendations and clinical practice guidelines [1]. Moreover, outcome and prognosis of myocarditis depend on its etiology, clinical presentation, and the stage of the disease at presentation to medical professionals [1, 2, 5]. The percentage of patients who develop persistent cardiac dysfunction or deteriorate acutely is unknown [1, 2] and treatment of many forms of myocarditis is symptomatic only [1]. Endomyocardial biopsy and immunohistochemical analysis are absolutely important, not only to confirm the diagnosis of myocarditis, but also to identify cases where specific therapy is available, such as giant cell myocarditis, eosinophilic myocarditis, or sarcoidosis [1]. Biventricular dysfunction at presentation has been reported as the main predictor of death, in need for assist device or transplantation [1, 2]. Myocarditis may clinically present in many different ways, ranging from mild symptoms of chest pain and palpitations only to life-threatening cardiogenic shock and ventricular arrhythmia [1] as presented in this case, making early diagnosis often very difficult. In many centers, endomyocardial biopsy is not generally performed, because of possible complications, which however are low in experienced teams [1, 5–7]. However, endomyocardial biopsy is of utmost importance in cases like this to confirm the diagnosis of myocarditis possibly by referring the patient to a specialized center. Furthermore, the underlying etiology of myocarditis has to be identified leading to a specific treatment in cases such as giant cell, eosinophilic myocarditis, or sarcoidosis, which need a different treatment [1, 5–7]. Our approach was successful in this rare case and although no infectious, immune-mediated, or toxic reason was detectable here, biopsy confirmed our treatment strategy and ensured not to have missed any specifically treatable cause. This is in accordance with current literature [1–10].

Fulminant myocarditis is believed to differ from a subacute or acute lymphocytic myocarditis in its mode of onset and the degree of hemodynamic compromise, but data is scarce in adult patients [1]. With this report we intend to emphasize that myocardial failure due to fulminant myocarditis may be reversible if treated with temporary extracorporeal life support being an important and life-saving tool to be considered in such severe cases. Nevertheless, further research in this field is needed to achieve improved outcome, such as reported in our case.
